# The Prevalence and Association of Depression and Anxiety With Multiple Sclerosis in Riyadh, Saudi Arabia: A Cross-Sectional Study

**DOI:** 10.7759/cureus.12389

**Published:** 2020-12-30

**Authors:** Ali Bahathig, Mohammed A Alblowi, Amna A Alhilali, Balqees S AlJasim, Manal Alhelow, Hamad Aldakheel, Nasser Alodayani, Narjes Hikri

**Affiliations:** 1 Psychiatry, College of Medicine, King Saud University, Riyadh, SAU; 2 Psychiatry, King Saud University, Riyadh, SAU; 3 Psychiatry, Alfarabi College, Riyadh, SAU; 4 College of Medicine, Al-Imam Muhammad Ibn Saud Islamic University, Riyadh, SAU

**Keywords:** anxiety, depression, neuropsychiatry, multiple sclerosis, quality of life, prevalence, saudi arabia

## Abstract

Introduction

Multiple sclerosis (MS) is often associated with depression and anxiety, with no clear prevalence, and the relationship between them is not fully understood.

Methods

In a cross-sectional study of 365 multiple sclerosis patients selected through a random sampling method from the MS society of Riyadh, Saudi Arabia, we collected data by self-administered questionnaires - the Patient Health Questionnaire (PHQ-9) and the Generalized Anxiety Disorder (GAD-7) questionnaire - and analyzed by descriptive and analytical statistics.

Results

Approximately 51.1% of participants had moderate or severe anxiety and 64% of them had depression among community-based multiple sclerosis patients according to Generalized Anxiety Disorder (GAD-7) questionnaire, and according to the PHQ-9 Arabic version about 28.85% showed mild to moderate depression.

The MS patients aged 53 to over 60 years showed higher levels of mild anxiety (76.32%) and higher levels of depression ranging from mild to moderate (53.61%) (P = 0.001). While the MS patients younger than 53 years showed moderate to severe anxiety (60.71%) and depression levels (62.32%). In different age group bad health status (37.6%) was associated with a higher prevalence of depression levels (P =< 0.001) and people who were widowed (22.38%), divorced (26%), and separated (37.82%) had significant depression levels (P = 0.017). In terms of anxiety, 54.58% of females had anxiety (P = 0.005), more older people showed anxiety (43.17%) (P = 0.026), and people with a bad general health state (26.38%) had anxiety (P = <0.001).

Conclusion

Among different types of MS patients, anxiety is more prevalent in the elder group (76%) of people and depression is more prevalent in young patients (62%).

## Introduction

The existence of a long-lasting incurable disease in a person’s life is a very traumatic event that requires special mental, social, and physical accommodation [1]. Multiple sclerosis (MS) is one of these major chronic illnesses.

MS is a neurodegenerative disease affecting the central nervous system (CNS) and leading to the damage of myelin and axons [2]. MS directly affects individuals in their productive time of life, limiting their work capacity, which results in major social and economic consequences [3].

The onset of MS is typically seen in individuals aged between 20 and 40 years. MS is seen in women two times more than in men [4].

MS appears as painless neuronal damage manifesting as vision impairment, double vision, limb weakness, unsteadiness of gait, and bowel or bladder symptoms [5]. The causative instigators for MS remain undetermined, but observational studies suggest genetic and environmental triggers influencing the pathophysiology of MS, which itself remains little understood, but is thought to be autoimmune in nature [6]. Diagnosis of MS is based on evaluation of disease dissemination through radiological and clinical aspects [7]. Current evidence indicates a notable increase in the prevalence and incidence of the disease, and recent studies have suggested a moderate-to-high prevalence in the Middle East region [8]. According to a meta-analysis, however, MS prevalence is low in Saudi Arabia (25/100,000) [8]. The clinical course was relapsing remission in 60.7%, progressive relapse in 20.2%, and primary progression in 19.1%. Weakness, followed by sensory impairment, was the two most common presenting symptoms [8].

In addition to the neurophysiological manifestations of the disease, many psychiatric and physical symptoms, such as fatigue and cognitive issues, may be present, causing increasing burden and suffering to patients and frustrating the identification of depression and anxiety [9,10]. Psychopathological signs and symptoms have been well known since MS was first systematically described [9]. Haussleiter et al. observed a marked memory weakness combined with pathological laughing and weeping, euphoria, mania, hallucinations, and depression [9]. These symptoms can be broadly divided into two categories: mood disorder and neurocognitive disorder. Additionally, indicative treatment of MS will in general focus more on the physical as opposed to the emotional outcome [10]. An estimated one in two people with MS will develop a major depression over the course of their lifetime [11]. A cross-sectional study conducted in Jeddah, Saudi Arabia, showed that depression and anxiety usually affect the patients' employment status and social functioning. More than 50% of the patients reported moderate to severe problems with depression, which indicates that depression negatively correlates with their quality of life [12].

Depression is an important public health problem affecting approximately 350 million people worldwide, gaining ground as the fourth leading cause of disease load [13]. Several chronic diseases were found to be associated with depression. A positive relationship between depression and some chronic diseases has been established in adult populations, especially in the developed countries [13].

## Materials and methods

We conducted a cross-sectional study of 365 adult patients affected by MS (132 males and 232 females), selected through a random sampling method from the MS society of Riyadh, Saudi Arabia. We asked participants to complete self-administered questionnaires over a period of four months (from March 2020 to June 2020). We calculated the sample size using the standard Single Proportion Formula at a confidence level of 95%, precision of 5%, and level of significance of 0.05, adding 10% to the original number to compensate for possible losses.

We included participants who were diagnosed with MS, older than 18 years of age, living in Riyadh, had not been diagnosed with psychiatric disease, and were not taking psychotropic medications.

We excluded participants who lived outside of Riyadh city, were known to have psychiatric diseases such as bipolar or schizophrenia, were taking psychotropic or anxiety medications, or did not fill the questionnaire completely.

Prior to data collection, we obtained an IRB approval from King Saud University Research Ethics Committee in Riyadh city, and explained the study objectives to the patients and obtained their voluntary consent before enrolling them in the study.

Questionnaires and scales

We asked patients to complete a self-administered questionnaire that included demographic variables (age, sex, marital status, education, and job), MS clinical features (type, onset, frequency of hospital admission due to relapse, pharmacotherapy, and health status compared to the previous year) and other variables, such as comorbidities and physical health. Patients completed scales, including the Patient Health Questionnaire (PHQ-9) and the Generalized Anxiety Disorder (GAD-7) questionnaire. We used the Arabic validated versions of both questionnaires [14]. The PHQ-9 is a brief, self-administered scale based on the nine Diagnostic and Statistical Manual of Mental Disorder (DSM-IV) criteria of assessing depression, in which a score of 10 is frequently suggested as the cut-off score [15]. The PHQ-9 has a sensitivity of 88% and a specificity of 88% for major depression; as such, it represents an appropriate tool to screen for depression among MS patients [16].

The GAD-7 is a brief, self-administered scale for diagnosing generalized anxiety disorder with a cut-off point of ≥ 10, and has 89% sensitivity and 82% specificity [17]. GAD-7 studies which have adopted this instrument further support its reliability and internal validity for use among MS patients [18].

Statistical analysis

We analyzed the data using the Statistical Package for Social Studies (SPSS v.22; IBM Corp., Armonk, NY, USA). We expressed continuous variables as mean ± standard deviation and categorical variables as percentages. We used the t-test and one-way ANOVA for continuous variables, and the Chi-square test for categorical variables. We considered a p-value of <0.05 statistically significant.

## Results

Using the Statistical Package for Social Studies (SPSS v.22; IBM Corp., Armonk, NY, USA), we analyzed the responses from 365 participants through their answers in an online survey targeting MS patients in Riyadh.

Demographic characteristics of the respondents

Respondents were mostly young (52.7%), with females representing twice the number of male participants (63.7%). More than half the respondents were married (58.2%), and most of them had university-level educations (59.9%), and the majority was unemployed (54.9%). Most of the participants responded that they were unaware of their type of MS (54.1%), while most of those who were aware were diagnosed with relapsing multiple sclerosis, the majority had a diagnosis duration of over two years (76.9%), and most reported having had a good to very good general state of health during those years (53.9%) (Table 1).

**Table 1:** Characteristics of the participants

		Number	(%)
Gender	Male	132	36.3
	Female	232	63.7
Age	18–25	54	14.8
	26–34	138	37.9
	35–43	109	29.9
	44–52	38	10.4
	53–60	19	5.2
	60+	6	1.6
Type of multiple sclerosis	Relapsing	120	33.0
	Primary progressive	24	6.6
	Secondary progressive	23	6.3
	Don't know	197	54.1
Duration of diagnosis	<2 y	84	23.1
	>2 y	280	76.9
Health state in general	Bad	16	4.4
	Acceptable	84	23.1
	Good	80	22.0
	Very good	116	31.9
	Excellent	68	18.7
Health state in comparison with previous year	Much worse than the previous year	15	4.1
	Somewhat worse than the previous year	68	18.7
	Almost the same	115	31.6
	Somewhat better	89	24.5
	Much better than the previous year	77	21.2
Educational level	Secondary or less	127	34.9
	University	218	59.9
	Master & PHD	19	5.2
Employment status	Free business	15	4.1
	Unemployed	200	54.9
	Employee full-time	132	36.3
	Employee part-time	17	4.7
Social status	Single	129	35.4
	Married	212	58.2
	Widow	3	.8
	Divorced	15	4.1
	Separated	5	1.4

The results showed that more than half of the participants had moderate or severe anxiety (51.1%) and depression (64%) among community-based MS patients in Riyadh, according to the Generalized Anxiety Disorder (GAD-7) questionnaire Arabic version, while 28.85% of participants showed mild to moderate depression, according to the PHQ-9 Arabic version (Table 2).

**Table 2:** Prevalence of depression and anxiety among community-based patients with multiple sclerosis

Disorder	Level	Number	(%)
Anxiety	0–4 minimal anxiety	73	20.1
	5–9 mild anxiety	103	28.3
	10–14 moderate anxiety	94	25.8
	15–21 severe anxiety	92	25.3
Depression	0–4 minimal depression	28	7.7
	5–9 mild depression	103	28.3
	10–14 moderate depression	107	29.4
	15–19 moderately severe depression	71	19.5
	20–27 severe depression	55	15.1

Data analysis revealed no significant difference among different types of multiple sclerosis according to the level of anxiety or depression (p = 0.591, 0.288, respectively) (Table 3).

**Table 3:** Prevalence of depression and anxiety among different types of multiple sclerosis

		Relapsing		Primary progressive		Secondary progressive		Don't know		P-value
Disorder	Level	Number	(%)	Number	(%)	Number	(%)	Number	(%)	
Anxiety	0–4 Minimal anxiety	25	20.83	8	33.33	4	17.39	36	18.46	0.591
	5–9 Mild anxiety	36	30.00	3	12.50	8	34.78	56	28.72	
	10–14 Moderate anxiety	33	27.50	6	25.00	7	30.43	48	24.62	
	15–21 Severe anxiety	26	21.67	7	29.17	4	17.39	55	28.21	
Depression	0–4 Minimal depression	11	9.17	0	0.00	4	17.39	13	6.60	
	5–9 Mild depression	41	34.17	5	20.83	4	17.39	53	26.90	0.288
	10–14 Moderate depression	29	24.17	12	50.00	7	30.43	59	29.95	
	15–19 Moderately severe depression	23	19.17	3	12.50	5	21.74	40	20.30	
	20–27 Severe depression	16	13.33	4	16.67	3	13.04	32	16.24	

The prevalence of depression and anxiety among different age groups differed significantly; older age groups showed higher levels of mild anxiety (76.32%), compared to younger age groups (60%) (P = 0.007); with depression, results were similar, showing that older age groups had higher levels of depression ranging from mild to moderate depression (53.61%) (P = 0.001), while younger age groups showed moderate to severe anxiety (60.71%) and depression levels (62.32%) (Table 4).

**Table 4:** Prevalence of depression and anxiety by age group

Disorder	Level	Age group												
		18–25		26–34		35–43		44–52		53–60		60+		
		Number	(%)	Number	(%)	Number	(%)	Number	(%)	Number	(%)	Number	(%)	P-value
Anxiety	0–4 Minimal anxiety	8	14.81	31	22.46	17	15.60	6	16.67	8	42.11	3	50.00	0.007*
	5–9 Mild anxiety	20	37.04	33	23.91	32	29.36	10	27.78	5	26.32	3	50.00	
	10–14 Moderate anxiety	14	25.93	48	34.78	22	20.18	9	25.00	1	5.26	0	0.00	
	15–21 Severe anxiety	12	22.22	26	18.84	38	34.86	11	30.56	5	26.32	0	0.00	
Depression	0–4 Minimal depression	5	9.26	9	6.52	6	5.50	3	7.89	1	5.26	4	66.67	0.001*
	5–9 mild depression	17	31.48	39	28.26	31	28.44	9	23.68	7	36.84	0	0.00	
	10–14 Moderate depression	18	33.33	40	28.99	33	30.28	8	21.05	7	36.84	1	16.67	
	15–19 Moderately severe depression	5	9.26	32	23.19	22	20.18	11	28.95	0	0.00	1	16.67	
	20–27 Severe depression	9	16.67	18	13.04	17	15.60	7	18.42	4	21.05	0	0.00	

The means of depression and anxiety grouped by the characteristics of participants differed significantly for depression, according to health state in general, with bad health status (37.6%) associated with a greater prevalence of depression (P = <0.001) and according to social status, with people who were widowed (22.38%), divorced (26%) and separated (37.82%) associated with significant depression (P = 0.017). For anxiety, there were significant differences according to gender, with females having higher anxiety (54.58%) (P = 0.005). Age also proved significant, with older people showing more anxiety (43.17%) (P = 0.026), and people with a bad general health state (26.38%) associated with higher levels of anxiety (P = <0.001). Other characteristics such as the type of disease, duration of diagnosis, education level, and employment status showed no significant differences (Table 5).

**Table 5:** Mean of depression and anxiety by characteristics of the participants * Significant p value

		Depression			Anxiety		
		Mean	SD	P-value	Mean	SD	P-value
Gender	Male	11.80	6.17	0.109	8.92	5.65	0.005*
	Female	12.87	6.09		10.72	5.84	
Age	18–25	12.15	6.65	0.166	9.98	5.65	0.026*
	26–34	12.47	5.86		9.73	5.57	
	35–43	12.65	5.97		10.87	5.95	
	44–52	13.92	6.58		11.17	5.95	
	53–60	11.32	6.37		7.89	6.72	
	60+	7.00	5.02		4.17	1.94	
Type of multiple sclerosis	Relapsing	11.80	6.06	0.503	9.55	5.36	0.501
	Primary progressive	13.17	5.26		9.79	5.76	
	Secondary progressive	12.43	5.84		9.39	5.25	
	Don't know	12.81	6.31		10.50	6.17	
Duration of diagnosis	<2 y	13.32	6.00	0.151	10.50	5.01	0.437
	>2 y	12.23	6.16		9.94	6.06	
Health state in general	Bad	18.31	5.31	<0.001*	14.00	6.88	<0.001*
	Acceptable	16.37	5.61		13.42	5.28	
	Good	13.08	6.28		10.03	6.00	
	Very good	10.92	5.05		9.31	5.16	
	Excellent	8.25	4.27		6.34	4.25	
Educational level	Secondary or less	13.10	5.92	0.246	10.94	6.28	0.072
	University	12.25	6.28		9.72	5.43	
	Masters & PHD	10.95	5.63		8.32	6.65	
Employment status	Free business	14.07	6.88	0.776	9.33	6.47	0.721
	Unemployed	12.43	6.05		10.06	5.79	
	Employee full-time	12.34	6.19		9.98	5.86	
	Employee part-time	12.71	6.34		11.53	5.83	
Social status	Single	12.39	5.86	0.017*	9.71	5.63	0.524
	Married	12.15	6.14		10.08	5.89	
	Widow	13.33	7.23		11.67	7.09	
	Divorced	15.00	7.00		11.73	6.47	
	Separate	20.60	3.36		13.00	6.16	

## Discussion

The association between MS and psychiatric disorders such as depression and anxiety is a complex relationship that has not yet been fully understood [19]. MS is a chronic disease associated with a considerable effect on the patient’s quality of life. Those who suffer from it require physical and psychological care [19]. Our results demonstrate the prevalence level of depression and anxiety among community-based multiple sclerosis patients in Riyadh, in which more than half of the participants had moderate or severe anxiety, while 28.85% of participants showed mild to moderate depression. We also found similar results in a study performed in the UK [20], describing a high frequency of MS patients with mild to moderate depression.

Some early studies have shown that there are differences in the levels of anxiety and depression based on genders and MS types. Anxiety was higher in people with relapsing remitting MS and depression was higher in people with primary progressive MS or secondary progressive MS [20]. There was little correlation between age or disease duration, with higher levels of anxiety in the group aged 15 to 24 years, and higher depression levels in the group aged 45 to 64 years and lower levels in the over 65 years age group [20]. On the other hand, some studies indicate there is no significant correlation between the rates of both disorders and any of the studied variables including duration of the disease, expanded disability status scale, age, gender, and level of education [21].

According to our results, there is no significant difference among different types of multiple sclerosis in terms of the level of anxiety or depression. This view was the opposite of that in a study published in 2012 [20], indicating that female patients with relapsing remitting MS were more anxious than males with this type of MS, and that a female with other types of MS and secondary progressive MS patients were more depressed than other types of MS patients, regardless of gender. Our result showed that most of the participants were unaware of the type of multiple sclerosis they had.

Most of the patients in our study were female and were aged between 26 and 34 years. Most of them were married. A previous study concluded that most MS patients’ relationships with partners, children and close friends were happy and healthy, factors which have an impact on mental health [22]. Being married was associated with a good prognosis in terms of disease progression [23]. These findings are consistent with our findings showing a significant difference in social status, in that people who were widowed, divorced, and separated had significant depressive symptoms.

Most of the participants had college degrees and the majority was unemployed, factors which could cause stress, as disability, gender and employment status exert potentially large effects on quality of life [24]. Furthermore, one report suggested that patients who were unemployed or housewives reported depressive symptoms more frequently than students or employed patients did [20].

An Iranian study demonstrated that depression is significantly associated with fatigue and older age, regardless of other factors. Moreover, most MS patients do not receive adequate treatment, despite the high prevalence of depression in MS [25]. The prevalence of anxiety and depression among MS patients is high and the relationship between economic status and depression and anxiety is significant [26]. In the current study, the prevalence of depression and anxiety differed among different age groups, with the older age groups showing higher levels of both mild anxiety and depression, on average. This finding is not consistent with the results of the previous study [19]. Our study also found a significant relationship between depression and the health state of MS patients, with depression correlating with a deteriorating health status.

Similar to the results of a study performed in 2012, the prevalence of possible depression was high during relapse, reducing gradually post-relapse [27]. Our approach differs from others in the literature since it focuses principally on community-based MS patients; we did not go to hospitals and gather information from there, but rather distributed the survey online for people to answer it, giving us a different perspective on the status of that population.

The limitations of our study

Some of the participants may have forgotten some information - for example, disease type - since their last visit to the doctor, which could have led to some recall bias. The time during which some of the answers were provided was a period of total lockdown in Riyadh city due to the COVID19 situation, which could have affected our results. While our survey was conducted from 25 March 2020 to 23 June 2020, the partial lockdown was implemented from 14 May 2020 to 22 May 2020 and the full lockdown was implemented from 23 May 2020 to 27 May 2020. The majority of the participants did not know what type of MS disorder they had, which could also have affected our results in terms of the correlation between the type of the disease and the anxiety or depression level. The results of the present study need to be evaluated carefully since we did not address the issue of social support and the side-effects of MS medications.

## Conclusions

With regard to the prevalence of depression and anxiety among community-based MS patients in Riyadh, our results indicate that more than half of the participants had moderate or severe anxiety (51.1%), while 64% of participants showed moderate or severe depression, and no significant difference in levels of anxiety or depression among different types of MS. Between different age groups, older age groups (aged 53 to over 60 years) reported higher levels of mild anxiety and higher levels of depression, ranging from mild to moderate depression, relative to levels in younger age groups. According to the characteristics of participants, people with a worse health status and differences in social status had a similar tendency for significant deviation towards depression and anxiety, females and middle-aged to older-aged patients deviating more significantly toward anxiety.

Researchers need to consider the type of medication used by patients that may exert deleterious effects on them and cause other side effects, including depression and anxiety, as well as the importance of social support for this group and the need for education of patients regarding various forms of MS, which can also have a significant impact on the quality of their psychosocial well-being.
